# Encapsulation of Ru(II) Polypyridine Complexes for Tumor-Targeted Anticancer Therapy

**DOI:** 10.34133/bmef.0024

**Published:** 2023-08-01

**Authors:** Johannes Karges

**Affiliations:** Faculty of Chemistry and Biochemistry, Ruhr-University Bochum, Universitätsstrasse 150, 44780 Bochum, Germany.

## Abstract

Ru(II) polypyridine complexes have attracted much attention as anticancer agents because of their unique photophysical, photochemical, and biological properties. Despite their promising therapeutic profile, the vast majority of compounds are associated with poor water solubility and poor cancer selectivity. Among the different strategies employed to overcome these pharmacological limitations, many research efforts have been devoted to the physical or covalent encapsulation of the Ru(II) polypyridine complexes into nanoparticles. This article highlights recent developments in the design, preparation, and physicochemical properties of Ru(II) polypyridine complex-loaded nanoparticles for their potential application in anticancer therapy.

## Introduction

In the developed world, cancer is one of the leading causes for death with about 19.3 million new cases and 10.0 million deaths recorded in 2020. These numbers are projected to surge in the upcoming decades, with an anticipated 28.4 million new cases annually by 2040 [1]. The prevalence of cancer has created a pressing need for the development of effective treatment methods. Traditional treatment modalities involve a combination of techniques whereby the tumor is removed in a surgical procedure and the patient is further treated by immunotherapy, radiotherapy, or chemotherapy. Since the discovery of cisplatin in the late 1960s, metal-based drugs have been extensively studied as chemotherapeutic agents. The biomedical mechanism of cisplatin is thought to be related to DNA damage, inhibition of replication and transcription, or a combination of both processes. One of the main factors contributing to the cytotoxicity of cisplatin is the formation of covalent cross-links when it interacts with DNA. The cytotoxicity of cisplatin is further amplified by overwhelming the cellular ability to repair the platinum–DNA adducts. Despite being one of the most widely used and effective chemotherapeutic drugs thus far, cisplatin is associated with severe side effects, including kidney damage, peripheral nerve damage, severe nausea, vomiting, and bone marrow suppression. These side effects, along with the development of tumor resistance, have limited the clinical use of cisplatin [2–7]. One promising alternative is the development of a new class of compounds such as ruthenium (Ru) complexes.

Over the last decades, the development of Ru complexes as chemotherapeutic agents has received increasing attention because of the generally non-toxic nature of these compounds and the presence of these metal complexes in variable oxidation states. Several Ru complexes have shown clinical potential as anticancer agents. Notably, the compounds imidazolium *trans*-[tetrachloro(dimethylsulfoxide) imidazole ruthenium(III)] (NAMI-A) and imidazolium *trans*-[tetrachloro(dimethylsulfoxide) imidazole ruthenium(III)] (KP1019), as well as its sodium salt KP1339, have advanced into clinical trials. NAMI-A, as the first Ru complex to undergo clinical trials, has demonstrated potent tumor growth inhibition effects on primary and secondary metastatic tumors in animal models. Its antitumor mechanism involves enhancing actin-dependent cell adhesion while reducing cell invasion and migration. This results in the disturbance of the communication of the cancer cells with the extracellular matrix. The phase 2 clinical study yielded unsatisfactory results in terms of drug activation against disease progression and adverse effects on patients. These outcomes limited its further clinical development [8–10]. Subsequently, KP1019, a structural analog of NAMI-A with a different mechanism of action on cancer cells, was introduced into clinical trials. KP1019 disrupts the redox balance inside the cancer cells, leading the inhibition of DNA synthesis and G2/M cell cycle arrest. These responses ultimately induce cell death by apoptosis. Despite these promising biological effects, the poor water solubility poses a limitation for further clinical advancement [11,12]. To overcome this limitation, the sodium salt of KP1019, known as KP1339, has been pursued as a drug candidate [13].

Besides chemotherapeutic agents, in recent years, considerable attention has been devoted to Ru(II) polypyridine complexes. This is largely due to their attractive biological properties and unique photophysical and photochemical properties, which can be fine-tuned by altering the number and nature of the polypyridyl ligands surrounding the Ru(II) metal center. On the basis of these properties, Ru(II) polypyridine complexes are extensively studied as photosensitizers for photodynamic therapy or photoactivated chemotherapy [14–28]. It is important to highlight that one of these compounds, namely, TLD-1433 is currently studied as a photosensitizer for photodynamic therapy in phase 2 clinical trials for the treatment of non-muscle invasive bladder cancer [29,30]. Despite their promising therapeutic profile, the vast majority of these complexes are associated with poor water solubility, non-specific biodistribution, lack of tumor-targeting properties, systemic toxicity, and, consequently, a low therapeutic index that limits their clinical application. Moreover, while the photosensitizer itself should be non-toxic in the absence of light, its exposure to light can result in cellular damage. Because of the strong light scattering of the skin and tissue during treatment and the challenge of precisely irradiating the tumor site, healthy surrounding tissue is also typically damaged in a photodynamic therapy treatment. To date, various types of delivery systems have been generated. In general, these systems can be categorized as active or passive pathways. In active tumor targeting, a particular molecule such as a signaling peptide [31–36], oligonucleotide [37–39], oligosaccharide [40,41], protein [42,43], receptor targeting moiety [44–49], or antibody [50,51] is employed to transport the therapeutic molecule. In passive tumor targeting, the leaky and highly permeable vasculature as well as poor lymphatic tissue characteristics of the tumor are targeted through selenium nanoparticles [52–54], silver nanoparticles [55], gold nanoparticles [56–58], silica nanoparticles [59–64], upconverting nanoparticles [65–67], carbon nanotubes [68–70], or metal–organic frameworks [71,72]. Notably, polymeric nanoparticles have not been mentioned here as these are in-depth described below. Despite the endeavors made, most of the aforementioned transportation systems suffer from drawbacks such as low water solubility, complicated preparation methods, high cost, or reduced therapeutic efficacy. To address these limitations, there is a pressing need to develop a drug delivery system that can selectively transport Ru(II) polypyridine complexes to its intended target.

## Advantages of the Encapsulation

Among other strategies, the encapsulation of metal complexes into polymeric materials, also referred to as nanomedicines, could present a viable solution to overcome the pharmacological limitations of the molecular therapeutic agents. Because of their unique features, such as small size, high surface area, surface chemistry, water solubility, and multifunctionality, polymeric materials are highly suitable for drug delivery purposes. By utilizing the abnormalities of the tumor vasculature, nanoparticles can accumulate in malignant tumors. This phenomenon is commonly referred to as the enhanced permeation and retention effect. Capitalizing on this, research efforts have been devoted to the incorporation of drugs into nanoparticles to overcome physical and pharmacokinetic limitations of molecular therapeutic agents [73–79].

Yu et al. [80] and Karges [81] have described a new research direction upon combination of material science with biosafety science termed as biosafety materials. The development of new materials that are able to influence biological or medicinal environments is expected to provide novel solutions for known and new medicinal problems and, therefore, actively shape research communities. Within this article, the encapsulation of anticancer agents into polymeric materials is systemically discussed.

## Encapsulation-Dependent Parameters

The encapsulation efficiency as well as biological and pharmacological properties of the formed nanoparticles are dependent on various factors. Some of the most important parameters are highlighted here.

• Particle size: The efficiency of targeted delivery of encapsulated Ru(II) polypyridine complexes to cancerous tissues is directly influenced by the size of the nanocarrier. Small, molecular Ru(II) polypyridine complexes can diffuse into the interstitial fluid, causing undesired side effects. To overcome this, efficient macromolecular delivery systems have been designed by exploiting the structural differences between tumorous and healthy tissues. For biological applications, the ideal diameter range for a carrier is 10 to 1,000 nm, but ideally, it should not exceed 300 nm to enable the enhanced permeation and retention effect and to ensure efficient passive targeting of tumor tissues [82,83].
• Particle charge: The stability and targeting efficiency of nanocarriers are directly influenced by their surface charge. Nanoparticles with a positively charged surface can typically easily enter cancerous cells through endocytosis. In contrast, nanoparticles with a neutral or negatively charged surface rely on specific interactions, resulting in reduced levels of non-specific adsorption of proteins and non-specific phagocytosis [84,85].
• Chemical structure of the polymer: The selection of a biocompatible polymer and the preparation method are crucial for ensuring the compatibility between the polymer and the metal complex. To achieve sufficient loading of the complex, a well-defined carrier structure is preferable to avoid phase separation and increase biological efficacy. Additionally, the integrity of the carrier nanostructure should be maintained post-encapsulation. Because of the charges of the Ru(II) polypyridine complexes, hydrophobic or electrostatic interactions between the polymer and the metal complex must be controlled [86,87].
• Biodegradability of the polymer: To ensure optimal performance of the polymeric nanocarriers for the Ru(II) polypyridine complex delivery, the biodegradability and composition of the polymer must be carefully controlled. For most applications, biodegradable polymers are required to regulate selective and specific release of the Ru complex. While nonbiodegradable polymers can improve properties such as stability and hydrophilicity, their elimination can occur without releasing the active compound. When biodegradable polymers are used, the surface-adsorbed Ru(II) polypyridine complex is released through initial hydrolysis of cleavable linkages, followed by slow polymer degradation over weeks to years to control payload release. Factors influencing the rate of Ru complex release include concentration gradient, mobility and diffusion within the nanocarrier, and polymer degradation rate. These properties are dependent on the composition, molecular weight, distribution, crystallinity, and chemical structure. Different degradation kinetics can be obtained depending on the regio- or stereoregularity of the polymer sequence in copolymers. Physical entrapment of the Ru(II) polypyridine complex is preferred over covalent conjugation as it maintains the integrity of the complex, but nanocarrier stability and preservation remain as challenges. A balance must be struck between too much stability, which can lead to poor release, and too little stability, which can result in premature disassembly or poor targeting efficiency [88,89].

## Polymeric Nanoparticles

In the 1980s, polymeric nanoparticles were reported for the first time as carriers for drug delivery [90,91]. These nanoscale particles are self-assembled usually in an aqueous solution from amphiphilic block copolymers. Spherical polymeric micelles typically have a diameter ranging from 10 to 100 nm [92]. However, their size can increase when proteins are adsorbed, leading to the formation of particles that are too large for renal excretion [93]. Apart from traditional spherical shapes, polymeric micelles can also self-assemble into flexible and cylindrical structures [94].

To achieve stable dispersion in aqueous environments, core–shell micelle architectures are typically obtained using diblock copolymers. The outer shell consists of hydrophilic blocks to protect the encapsulated Ru(II) polypyridine complex from adsorption of biomolecules during circulation or interaction with cellular membranes. The inner core, made up of the hydrophobic block, is stabilized by hydrophobic interactions and serves as a reservoir for the encapsulating hydrophobic Ru(II) polypyridine complexes. Amphiphilic diblock copolymers with longer hydrophilic segments are used to form spherical micelles. However, the limited kinetic stability can pose a challenge because they exist in a dynamic equilibrium between the self-assembled micelle and the bulk phase. To enhance the targeting specificity toward diseased tissues or organelles, the surface of a polymer can be functionalized with recognition motifs [95–97].

Polymer-based nanocarriers are widely employed as the preferred drug delivery system because of their facile synthesis, diverse composition, architecture, functionalization, and ability to degrade in physiological media. With a wide range of polymer architectures available, these are among the most promising drug delivery systems, including polymeric micelles, nanogels, vesicles, dendrimers, and nanoparticles. There are 2 strategies for encapsulating drugs within a polymer matrix: (a) physical encapsulation, which relies on noncovalent interactions between the drug and the polymer matrix, and (b) covalent encapsulation, which involves the covalent conjugation of the drug to the polymer [98–100]. Subsequently, these types of encapsulations are separately discussed.

### Physical encapsulation into polymeric nanoparticles

Polymeric nanoparticles are created by self-assembling amphiphilic polymers, which form a hydrophobic core and a hydrophilic shell to encapsulate therapeutic compounds and stabilize the interface between the core and the aqueous medium. As the predominant method, therapeutic compounds are physically encapsulated with amphiphilic polymers because of their facile synthesis and easy optimization into nanoparticles with tailored properties. The most widely used biocompatible and biodegradable polymers are aliphatic polyesters, such as the Food and Drug Administration-approved polylactide and poly(D,L-lactide-co-glycolide). Polylactide and poly(D,L-lactide-co-glycolide) break down into non-toxic acidic products, specifically lactic acid and glycolic acid, which can be metabolized to produce harmless by-products such as carbon dioxide and water. Despite these promising properties, this method of encapsulation is associated with several limitations including (a) the burst release, which involves the sudden release of the drug; (b) difficulties in encapsulating drugs that are poorly miscible with the polymer matrix; and (c) poor drug loading, necessitating a high concentration of the nanoparticles to achieve a therapeutic effect [101–103].

**Fig. 1.**
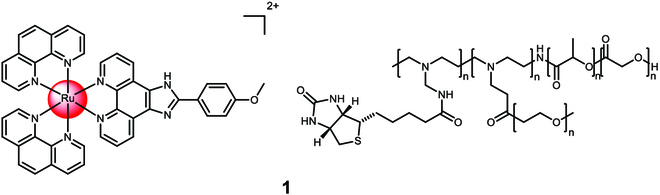
Structure of the physical encapsulation of Ru(1,10-phenanthroline)_2_(2-(4-methoxyphenyl)imidazo[4,5-f][1,10]phenanthroline)]^2+^ with poly(D,L-lactide-co-glycolide), which was previously functionalized with biotin and polyethylene glycol, into 1. The counterions were omitted for clarity.

Chan et al. [104] described the encapsulation of the anticancer agent [Ru(1,10-phenanthroline)_2_(2-(4-methoxyphenyl)imidazo[4,5-f][1,10]phenanthroline)]^2+^ in poly(D,L-lactide-co-glycolide) nanoparticles using the nanoprecipitation technique. To improve their pharmacological properties, the nanoparticles were coated with polyethylenimine that was previously prepared from biotin and polyethylene glycol (Fig. 1). The resulting nanoparticle **1** had a diameter of 150 nm and showed tumor-targeting capabilities, particularly toward cancer cells that overexpressed sodium multivitamin transporter receptors. The researchers confirmed the spherical shape of the nanoparticles using transmission electron microscopy. The nanoparticles demonstrated high stability in cell media and human plasma. In comparison to the molecular metal complex or unmodified Ru(II) polypyridine complex nanoparticles, the loaded nanoparticle **1** exhibited 3- to 4-fold higher toxicity against human hepatocellular carcinoma cells (HepG2). In a xenograft mouse model, the biodistribution analysis revealed that the loaded nanoparticles accumulated primarily in the liver and tumor, while the molecular metal complex was distributed throughout the body, indicating the potential of the nanoparticle formulation to improve the biodistribution of the metal complex in the animal model.

**Fig. 2.**
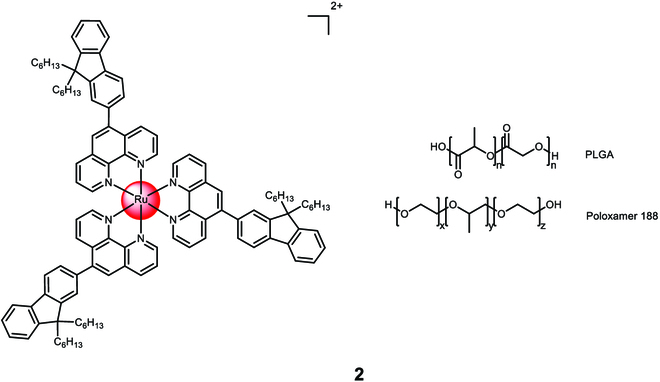
Structure of the physical encapsulation of the Ru(II) polypyridine complex with a 50:50 mixture of poly(D,L-lactide-co-glycolide) and Poloxamer 188 into 2. The counterions were omitted for clarity.

Bœuf et al. [105] developed nanoparticles that encapsulate 5-substituted-1,10-phenanthroline functionalized Ru(II) polypyridine complexes using poly(D,L-lactide-co-glycolide). The nanoparticles were generated through nanoprecipitation in the presence of Poloxamer 188 and acid-terminated poly(D,L-lactide-co-glycolide) (Fig. 2). The drug loading efficiency was approximately 1%. The researchers successfully obtained spherical nanoparticle **2** with a size of 100 nm and low polydispersity index. Upon irradiation, around 50% of the Ru(II) polypyridine complex payload was released within 2 days, while only 10% was released after 6 days of incubation in the dark. The nanoparticles showed minimal toxicity in the dark, but when exposed to white light irradiation (30 min, 17 mW/cm^2^), the entire cell population of glioma (C6) cells was eliminated at a concentration of 0.1 μM.

**Fig. 3.**
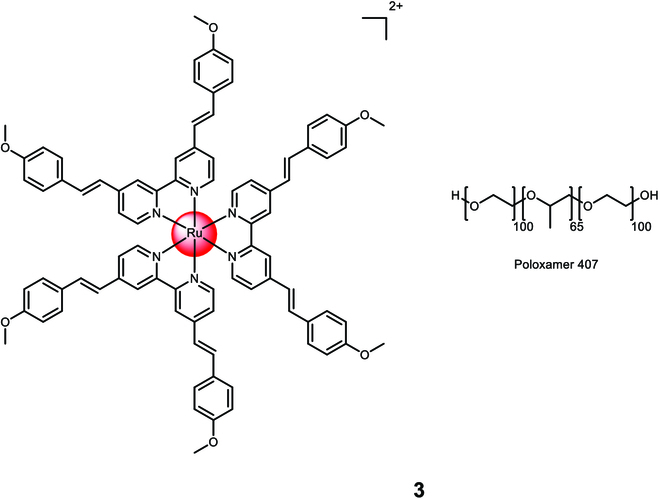
Structure and transmission electron microscopy image of the physical encapsulation of [Ru((*E*,*E*′)-4,4′-Bis[*p*-methoxystyryl]-2,2′-bipyridine)_3_]^2+^ with Poloxamer 407 into 3. The counterions were omitted for clarity.

Karges et al. [106] performed a study on the encapsulation of [Ru((*E*,*E*′)-4,4′-Bis[*p*-methoxystyryl]-2,2′-bipyridine)_3_]^2+^ with Poloxamer-407 (Pluronic F-127) to form nanoparticle **3** (Fig. 3). The average size of the nanoparticles was measured to be between 53 and 162 nm, and they exhibited a spherical shape as observed through transmission electron microscopy. The encapsulated Ru(II) polypyridine complex displayed the ability to generate singlet oxygen when irradiated at 500 nm. Because of the ligand’s high lipophilicity, the Ru(II) polypyridine complex itself had poor water solubility. However, after encapsulation, the resulting nanoparticles showed high water solubility. The nanoparticles were non-toxic in the absence of light. When exposed to irradiation at 500 nm for 16.7 min with an energy density of 10 J/cm^2^, they exhibited cytotoxic effects against human cervical carcinoma (HeLa) cells. The concentration at which the nanoparticles caused a cytotoxic effect, known as the CC_50_ value, ranged from 93 to 261 μM based on the loading of the Ru(II) polypyridine complex.

**Fig. 4.**
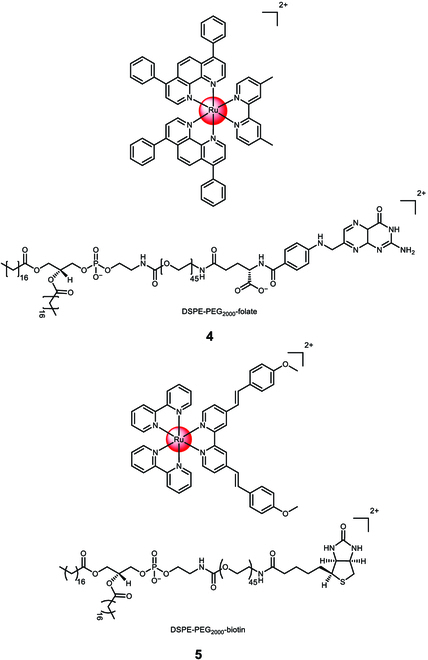
Structure of the physical encapsulation of the Ru(II) polypyridine complex [Ru(4,7-diphenyl-1,10-phenanthroline)_2_(4,4′-dimethyl-2,2′-bipyridine)]^2+^ with 1,2-distearoyl-sn-glycero-3-phosphoethanolamine-*N*-[folate(polyethylene glycol)-2000][ammonium salt] (DSPE-PEG_2000_-folate) into 4 or the Ru(II) polypyridine complex [Ru(2,2′-bipyridine)_2_((*E*,*E*′)-4,4′-Bis[*p*-(*N*,*N*-methoxy)styryl]-2,2′-bipyridine)]^2+^ with 1,2-distearoyl-sn-glycero-3-phosphoethanolamine-*N*-[biotin(polyethylene glycol)-2000][ammonium salt] (DSPE-PEG_2000_-biotin) into 5. The counterions were omitted for clarity.

Karges et al. [107] conducted a study on the encapsulation of a Ru(II) polypyridine complex, [Ru(4,7-diphenyl-1,10-phenanthroline)_2_(4,4′-dimethyl-2,2′-bipyridine)]^2+^, using a commercially available polymer 1,2-distearoyl-sn-glycero-3-phosphoethanolamine-*N*-[folate(polyethylene glycol)-2000][ammonium salt] (DSPE-PEG_2000_-folate) (Fig. 4). The resulting nanoparticle **4** had an average size of 122 nm. The molecular complex itself had an undesired cytotoxic effect in the dark with varying cytotoxicity across different cell lines (CC_50,dark_ = 28.8 to 3.1 μM). However, the formulation of the complex into nanoparticles overcame this limitation as the nanoparticles were found to be non-toxic in the absence of light. Upon irradiation at 480 nm (10 min, 3.1 J/cm^2^) or 595 nm (60 min, 11.3 J/cm^2^), the nanoparticles demonstrated phototoxicity in the low micromolar range in 2-dimensional monolayer human ovarian carcinoma (A2780) cancer cells (CC_50,dark_ > 100 μM, CC_50,480nm_ > 2.64 ± 0.33 μM, CC_50,595nm_ > 3.51 ± 0.64 μM) and in 3-dimensional A2780 multicellular tumor spheroids (CC_50,dark_ > 100 μM, CC_50,480nm_ > 8.16 ± 0.87 μM, CC_50,595nm_ > 9.62 ± 0.93 μM). The nanoparticles also exhibited effectiveness against drug-resistant cancer cell lines, indicating their ability to overcome drug resistance. Inductively coupled plasma mass spectrometry studies confirmed that the nanoparticles accumulated 8 times more in cancer cells that overexpressed folate receptors, thus validating their cancer-targeting effect [108].

Karges et al. [109] conducted a study on the physical encapsulation of a Ru(II) polypyridine complex, [Ru(2,2'-bipyridine)_2_((*E*,*E*′)-4,4′-Bis[*p*-(*N*,*N*-methoxy)styryl]-2,2′-bipyridine)]^2+^, using a commercially available polymer 1,2-distearoyl-sn-glycero-3-phosphoethanolamine-*N*-[biotin(polyethylene glycol)-2000][ammonium salt] (DSPE-PEG_2000_-biotin) **5** (Fig. 4). Through confocal laser scanning microscopy transfection assay and the determination of the metal content using inductively coupled plasma mass spectrometry, the researchers verified the preferential accumulation of the nanoparticles in cancer cells that overexpressed sodium multivitamin transporters. Quantification demonstrated approximately 20 times higher accumulation in sodium multivitamin transporter-overexpressed adenocarcinomic human alveolar basal epithelial (A549) cancer cells compared to noncancerous human lung fibroblast cells. The nanoparticles exhibited enhanced cytotoxicity against cancerous A549 cells (CC_50,500nm_ > 3.2 ± 0.1 μM, CC_50,800nm_ > 3.2 ± 0.2 μM) compared to non-cancerous human lung fibroblast cells (CC_50,500nm_ > 48.1 ± 3.6 μM, CC_50,800nm_ > 48.2 ± 4.0 μM) when exposed to 1-photon irradiation (500 nm, 11 mW/cm^2^, 6.0 J/cm^2^) or 2-photon irradiation (800 nm, 0.29 mW/cm^2^, 80 MHz, 100 fs, 10.1 J/cm^2^). The nanoparticles were non-toxic in the absence of light (CC_50,dark_ > 494.7 μM). In a A549 tumor-bearing mouse model, the nanoparticles exhibited an 8.7 times higher accumulation in the tumor compared to the unformulated complex when the same amount of the Ru(II) polypyridine complex was intravenously injected, demonstrating their cancer-targeting capabilities. Upon exposure to clinically relevant 1-photon (500 nm, 11 mW/cm^2^, 6.0 J/cm^2^) or 2-photon (800 nm, 50 mW, 1 kHz, pulse width of 35 fs, 5 s/mm) excitation, the nanoparticles nearly completely eradicated the tumor within the mouse model.

**Fig. 5.**
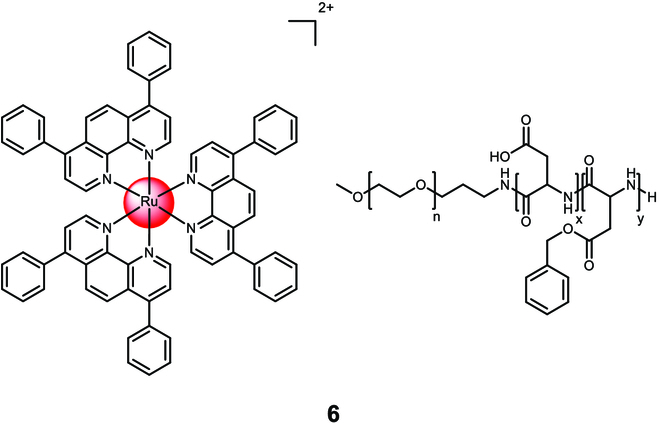
Structure of the physical encapsulation of the Ru(II) polypyridine complex [Ru(4,7-diphenyl-1,10-phenanthroline)_3_]^2+^ with polyethylene glycol-block-poly(L-aspartate) copolymers into 6. The counterions were omitted for clarity.

Dickerson et al. [110] developed cross-linked nanoassemblies using polyethylene glycol-block-poly(L-aspartate) copolymers as a nanogel delivery platform for the Ru(II) polypyridine complex [Ru(4,7-diphenyl-1,10-phenanthroline)_3_]^2+^ (Fig. 5). The nanoassembly **6** had an average diameter of 19 nm and achieved a drug loading efficiency of up to 20%. The release rate of the metal complex was influenced by its hydrophobicity and the ionic strength of the solution, while pH changes had minimal impact. This suggests that the drug release can be controlled and tailored for specific applications. In terms of cytotoxicity, the nanoparticles and the unformulated Ru(II) polypyridine complex showed similar profiles in the absence of light (CC_50,dark,complex_ = 0.6 ± 1.1 μM, CC_50,dark,nanoparticle_ > 0.6 ± 1.2 μM) and when exposed to irradiation (>400 nm) (CC_50,light,complex_ = 0.1 ± 1.0 μM, CC_50,light,nanoparticle_ > 0.1 ± 1.1 μM) against A549 cells.

### Covalent encapsulation into polymeric nanoparticles

To formulate polymer–drug conjugates into nanosized constructs, covalent encapsulation is utilized. This approach involves linking the drug covalently to a hydrophilic polymer [111]. The connectivity and position of the modification of the Ru(II) polypyridine complex onto the polymer such as a terminal group or the polymeric backbone determines its biological properties and the specific synthetic strategies used for its preparation.

**Fig. 6.**
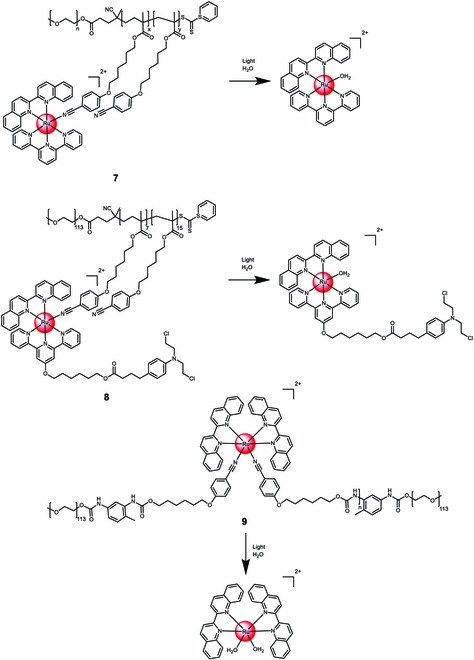
Structure and drug release upon exposure to irradiation of the covalent encapsulation of [Ru(2,2′:6′,2″-terpyridine)(2,2′-biquinoline)(H_2_O)]^2+^ or [Ru(2,2′-biquinoline)_2_(H_2_O)]^2+^ with polyethylene glycol-block-poly(6-(4-cyanophenoxy) hexyl methacrylate) into 7 to 9. The counterions were omitted for clarity.

Sun et al. [112] developed amphiphilic block copolymers containing a Ru(II) polypyridine complex, enabling high drug loading and self-assembly into sub-150-nm nanostructures. The [Ru(2,2′:6′,2′′-terpyridine)(2,2′-biquinoline)(H_2_O)]^2+^ complex was coordinated to a preformed polymer, polyethylene glycol-block-poly(6-(4-cyanophenoxy)hexyl methacrylate) **7** (Fig. 6), which could release the therapeutic Ru(II) complex upon light exposure. Subsequently, Sun et al. [113] synthesized a polymeric material where the Ru(II) polypyridine complex was linked to the anticancer drug chlorambucil via ester bond formation **8** (Fig. 6). When dispersed in water, the amphiphilic polymer self-assembled into nanoparticles, measuring around 15 nm in diameter. In the dark, the nanoparticles exhibited no toxicity toward HeLa cells, both in normoxic and hypoxic environments. However, when exposed to light (56 nm, 60 J/cm^2^), the nanoparticles displayed cytotoxicity, with an effective concentration (EC_50_) of approximately 25 μg/ml under both normoxic and hypoxic conditions. Using a similar approach, the authors coordinated the Ru(II) polypyridine complex [Ru(2,2′-biquinoline)_2_(H_2_O)]^2+^ to an ABA triblock copolymer (polyethylene glycol-block-poly(6-(4-cyanophenoxy)hexyl methacrylate)) **9** (Fig. 6). Upon light exposure, the polymeric chains and therapeutic agents were released and singlet oxygen was generated. The nanoparticles were non-toxic in the dark (CC_50,dark_ > 150 μg/ml) but exhibited high cytotoxicity (CC_50,light_ ~ 25 μg/ml) against HeLa, HepG2, and human prostate cancer (PC3) cells upon irradiation (656 nm, 50 mW/cm^2^, 30 min). In a HeLa tumor-bearing mouse model, the nanoparticles selectively accumulated in the tumor upon intravenous injection. While not affecting tumor growth in the dark, intravenous injection combined with irradiation (655 nm, 0.2 W/cm^2^, 10 min) resulted in significant tumor growth inhibition [114]. On the basis of this concept, Zeng et al. [115] have prepared a dual-responsive Pt(IV)/Ru(II) bimetallic polymer that could self-assemble into nanoparticles. The nanoparticles were able interact in cancer cells through a combination of cancer-activated chemotherapy and photodynamic therapy. Promisingly, the nanomaterial demonstrated to nearly fully eradicate cisplatin-resistant tumors in a patient-derived xenograft model.

**Fig. 7.**
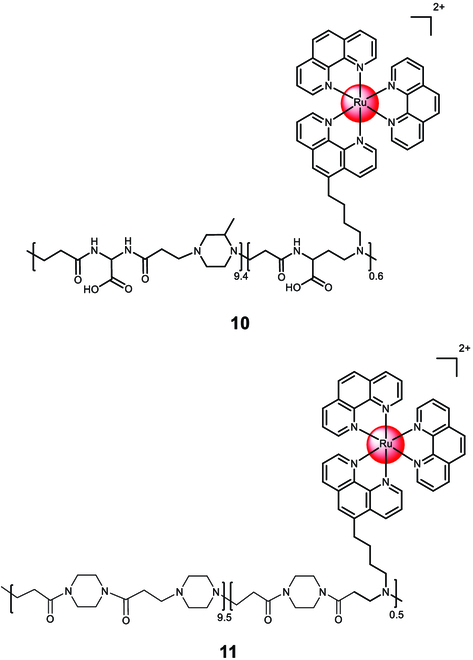
Structure of the covalent encapsulation of [Ru(1,10-phenanthroline)_3_]^2+^ with a zwitterionic polyamidoamine polymer into the nanoparticle 10 or a cationic polyamidoamine polymer into the nanoparticle 11. The counterions were omitted for clarity.

Maggioni et al. [116] have polymerized polyamidoamine chains to 1,10-phenanthroline that were further coordinated to Ru(II) polypyridine center **10** (Fig. 7). The resulting polymeric material self-assembled into nanoparticles with an average diameter of ~20 nm. The zwitterionic nature of the polymeric chain in an aqueous solution and stability studies in the presence of cysteine suggest its suitability for biological applications. The nanoparticles were readily internalized into HEK-293 cells through endocytosis or micropinocytosis, accumulating in vesicular compartments of the cytoplasm. Subsequently, Mascheroni et al. [117] incorporated the Ru(II) polypyridine complex [Ru(1,10-phenanthroline)_3_]^2+^ into a polyamidoamine polymer **11** (Fig. 7). The resulting polymeric material self-assembled into nanoparticles with an average diameter of ~10 nm. In an aqueous solution, the cationic nature of the polymer distinguished it from the analogous polymer **10**. Both **10** and **11** efficiently generated singlet oxygen upon light exposure. The zwitterionic polymeric nanoparticle **10** was non-toxic in the dark and under light irradiation (CC_50,dark/light_ > 50 μM) against HeLa cells. In contrast, the cationic polymeric nanoparticle **11** was non-toxic in the dark (CC_50,dark_ > 5 μM) but exhibited low micromolar cytotoxicity (CC_50,light_ = 0.7 μM) against HeLa cells upon irradiation with visible light (400 to 800 nm, 40 min, 23.7 mW/cm^2^).

**Fig. 8.**
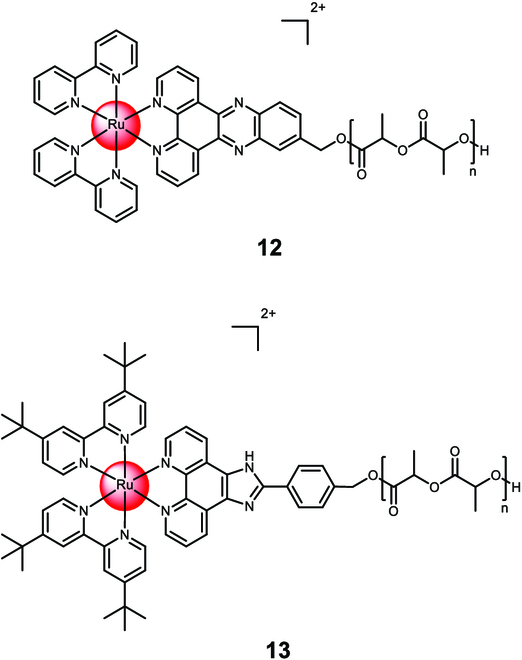
Structure of the covalent encapsulation of [Ru(2,2′-bipyridine)_2_(dipyrido[3,2-a:2′,3′-c]phenazin-7-hydroxymethyl)]^2+^ with lactide polymer into the nanoparticle 12 or the Ru(II) polypyridine complex [Ru(2,2′-bipyridine)_2_(4-hydroxymethyl-phenyl-1*H*-imidazo-1,10-phenanthroline)]^2+^ with lactide polymer into the nanoparticle 13. The counterions were omitted for clarity.

Soliman et al. [118] conjugated [Ru(2,2′-bipyridine)_2_(dipyrido[3,2-a:2′,3′-c]phenazin-7-hydroxymethyl)]^2+^ to lactide **12** via ring-opening polymerization (Fig. 8). These conjugates were found to self-assemble into nanoparticles and generate singlet oxygen upon irradiation. The nanoparticles showed enhanced cellular internalization compared to the free metal complex. They were non-toxic in the dark (CC_50,dark_ > 100 μM) but exhibited cytotoxicity upon irradiation (480 nm, 10 min, 3.21 J/cm^2^) against HeLa cells (CC_50,480nm_ = 16.7±4.3 μM). In another study, the Ru(II) polypyridine complex [Ru(2,2′-bipyridine)_2_(4-hydroxymethyl-phenyl-1*H*-imidazo-1,10-phenanthroline)]^2+^ was conjugated to lactide **13** (Fig. 8). Within 48 h of incubation under physiological conditions, nearly the whole payload of the Ru(II) polypyridine complexes was released. The nanoparticles were non-toxic after 48 h of incubation (CC_50,48h_ > 100 μM) but showed cytotoxicity after 72 h of incubation (CC_50,72h_ = 35.4 ± 2.9 μM) against A2780 cells. Studies inside a A2780 tumor-bearing mouse model showed a highly increased accumulation inside the tumorous tissue in comparison to the unformulated Ru(II) polypyridine complex. Despite these preliminary promising properties, **12** showed a negligible tumor growth inhibition effect [119].

## Liposomes

Liposomes are small vesicles ranging from nanosized to microsized, which contain an aqueous core enveloped by a phospholipid bilayer. They have made a significant breakthrough as a nanomedicine delivery system, becoming the first to transition from theory to clinical application, thus establishing them as a well-established technological platform with extensive clinical acceptance [120,121].

**Fig. 9.**
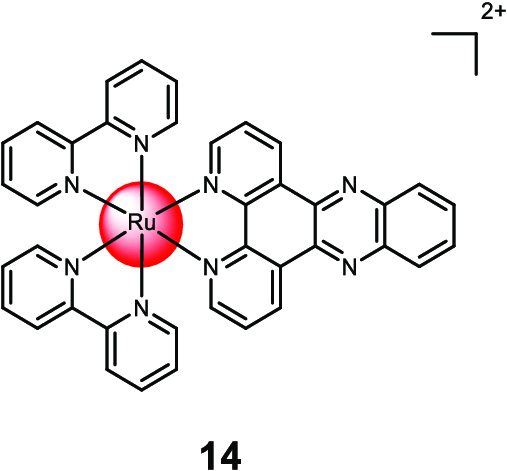
Structure of the encapsulation of [Ru(2,2′-bipyridine)_2_(dipyrido[3,2-a:2′,3′-c]phenazin)]^2+^ 14 with liposomes. The counterions were omitted for clarity.

Shen et al. [122] encapsulated the Ru(II) polypyridine complex [Ru(2,2′-bipyridine)_2_(dipyrido[3,2-a:2′,3′-c]phenazin)]^2+^**14** into liposomes composed of dipalmitoylphosphatidylcholine, polyethylene glycol-modified phospholipid, and cholesterol (Fig. 9). The liposomes exhibited enhanced cellular uptake compared to the free metal complex. While the liposomes without the metal complex were non-toxic, the Ru(II) polypyridine complex-loaded liposomes showed cytotoxicity in the micromolar range (CC_50_ ~ 4 μM) against breast cancer (MDA-MB-231) cells. Further investigation revealed that the liposomes induced DNA damage, leading to apoptosis. In an MDA-MB-231 tumor-bearing mouse model, the Ru(II) polypyridine complex-loaded liposomes selectively accumulated in the tumor tissue and significantly reduced tumor growth.

**Fig. 10.**
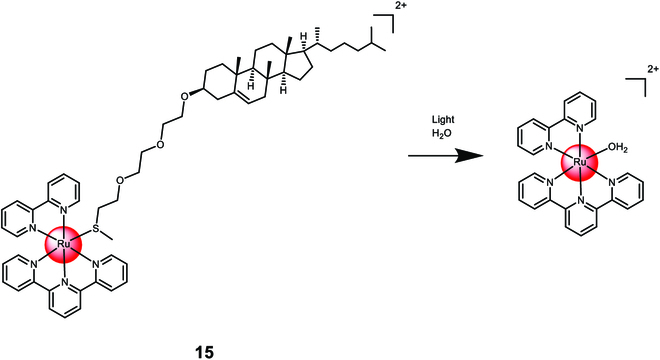
Structure and drug release upon exposure to irradiation of the covalent encapsulation of the Ru(II) polypyridine complex [Ru(2,2′:6′,2″-terpyridine)(2,2′-bipyridine)(thioether-cholestanol)]^2+^ 15 with liposomes. The counterions were omitted for clarity.

Askes et al. [123] developed liposomes encapsulating Ru(II) polypyridine complexes [Ru(2,2′:6′,2″-terpyridine)(2,2′-bipyridine)(thioether-cholestanol)]^2+^**15** (Fig. 10). A hybrid ligand combining thioether and cholestanol enabled coordination with the metal center. Negatively charged or neutral lipids were used for the liposome membranes. Under irradiation, the monodentate thiol ligand was released. To treat deep-seated or large tumors, the researchers combined the Ru(II) polypyridine complex-loaded liposomes with triplet–triplet annihilation upconversion liposomes. These upconversion liposomes could be excited with near-infrared light at 630 nm and emit blue light to trigger the photodissociation of the Ru(II) polypyridine complex.

## Summary and Perspectives

Extensive research has been conducted on the application of Ru(II) polypyridyl complexes for anticancer therapy. However, their low cellular uptake and lack of specificity for cancer cells and tumors have initiated the necessity of the development of nanomaterials that incorporate Ru(II) polypyridine complexes. The encapsulation of these metal complexes through into nanoparticles is a promising strategy for overcoming the pharmacological limitations of molecular agents and provides cancer selectivity. The development of nanomaterials loaded with Ru(II) polypyridine complexes is still in its early stages, and much remains to be understood. Various nanoplatform constructs are available for incorporating these metal complexes depending on the intended application. Some potential areas of focus for future studies include:

• Optimization of nanoparticle design: There is a need to optimize the design of nanoparticles to improve their efficiency and effectiveness in delivering Ru(II) polypyridine complexes to cancer cells. This could involve tailoring the size, shape, surface charge, and surface functionalization of nanoparticles to enhance their cellular uptake and targeting.
• Evaluation of toxicity and biodistribution: It is essential to investigate the toxicity and biodistribution of Ru(II) polypyridine complex-loaded nanoparticles to ensure their safety and efficacy for clinical use. Preclinical studies can provide valuable insights into the pharmacokinetics, pharmacodynamics, and potential adverse effects of these nanoparticles.
• Development of combination therapies: Combining Ru(II) polypyridine complex-loaded nanoparticles with other cancer therapies, such as chemotherapy, radiation therapy, and immunotherapy, could enhance their therapeutic efficacy and overcome resistance to treatment. This approach could also reduce the dosage of each therapy, minimizing side effects and improving patient outcomes.
• Translation to clinical applications: Ultimately, the development of Ru(II) polypyridine complex-loaded nanoparticles needs to be translated into clinical applications to benefit patients with cancer. This involves rigorous testing in clinical trials to demonstrate their safety and efficacy, as well as regulatory approval for clinical use.

Overall, the development of Ru(II) polypyridine complex-loaded nanoparticles has the potential to revolutionize cancer therapy by providing targeted and multimodal treatments with reduced side effects. Continued research and development in this area could lead to significant advances in the field of oncology.

## References

[1] Sung H, Ferlay J, Siegel RL, Laversanne M, Soerjomataram I, Jemal A, Bray F. Global cancer statistics 2020: GLOBOCAN estimates of incidence and mortality worldwide for 36 cancers in 185 countries. CA Cancer J Clin. 2021;71(3):209–249. 33538338 10.3322/caac.21660

[2] Dilruba S, Kalayda GV. Platinum-based drugs: Past, present and future. Cancer Chemother Pharmacol. 2016;77(6):1103–1124. 26886018 10.1007/s00280-016-2976-z

[3] Zhang C, Xu C, Gao X, Yao Q. Platinum-based drugs for cancer therapy and anti-tumor strategies. Theranostics. 2022;12(5):2115–2132. 35265202 10.7150/thno.69424 PMC8899578

[4] Oun R, Moussa YE, Wheate NJ. The side effects of platinum-based chemotherapy drugs: A review for chemists. Dalton Trans. 2018;47(19):6645–6653. 29632935 10.1039/c8dt00838h

[5] Rottenberg S, Disler C, Perego P. The rediscovery of platinum-based cancer therapy. Nat Rev Cancer. 2021;21(1):37–50. 33128031 10.1038/s41568-020-00308-y

[6] Johnstone TC, Suntharalingam K, Lippard SJ. The next generation of platinum drugs: Targeted Pt(II) agents, nanoparticle delivery, and Pt(IV) prodrugs. Chem Rev. 2016;116(5):3436–3486. 26865551 10.1021/acs.chemrev.5b00597 PMC4792284

[7] Xu Z, Wang Z, Deng Z, Zhu G. Recent advances in the synthesis, stability, and activation of platinum(IV) anticancer prodrugs. Coord Chem Rev. 2021;442:Article 213991.

[8] Rademaker-Lakhai JM, van den Bongard D, Pluim D, Beijnen JH, Schellens JHM. A phase I and pharmacological study with imidazolium-trans-DMSO-imidazole-tetrachlororuthenate, a novel ruthenium anticancer agent. Clin Cancer Res. 2004;10(11):3717–3727. 15173078 10.1158/1078-0432.CCR-03-0746

[9] Bergamo A, Messori L, Piccioli F, Cocchietto M, Sava G. Biological role of adduct formation of the ruthenium(III) complex NAMI-A with serum albumin and serum transferrin. Investig New Drugs. 2003;21(4):401–411. 14586207 10.1023/a:1026243000320

[10] Alessio E, Mestroni G, Bergamo A, Sava G. Ruthenium antimetastatic agents. Curr Top Med Chem. 2004;4:1525–1535. 15579094 10.2174/1568026043387421

[11] Hartinger CG, Jakupec MA, Zorbas-Seifried S, Groessl M, Egger A, Berger W, Zorbas H, Dyson PJ, Keppler BK. KP1019, a new redox-active anticancer agent—Preclinical development and results of a clinical phase I study in tumor patients. Chem Biodivers. 2008;5(10):2140–2155. 18972504 10.1002/cbdv.200890195

[12] Hartinger CG, Zorbas-Seifried S, Jakupec MA, Kynast B, Zorbas H, Keppler BK. From bench to bedside—Preclinical and early clinical development of the anticancer agent indazolium trans-[tetrachlorobis(1H-indazole)ruthenate(III)] (KP1019 or FFC14A). J Inorg Biochem. 2006;100(5):891–904. 16603249 10.1016/j.jinorgbio.2006.02.013

[13] Heffeter P, Böck K, Atil B, Reza Hoda MA, Körner W, Bartel C, Jungwirth U, Keppler BK, Micksche M, Berger W, et al. Intracellular protein binding patterns of the anticancer ruthenium drugs KP1019 and KP1339. J Biol Inorg Chem. 2010;15(5):737–748. 20221888 10.1007/s00775-010-0642-1 PMC3371400

[14] Gill MR, Thomas JA. Ruthenium(ii) polypyridyl complexes and DNA—From structural probes to cellular imaging and therapeutics. Chem Soc Rev. 2012;41(8):3179–3192. 22314926 10.1039/c2cs15299a

[15] Heinemann F, Karges J, Gasser G. Critical overview of the use of Ru(II) polypyridyl complexes as photosensitizers in one-photon and two-photon photodynamic therapy. Acc Chem Res. 2017;50(11):2727–2736. 29058879 10.1021/acs.accounts.7b00180

[16] Zeng L, Gupta P, Chen Y, Wang E, Ji L, Chao H, Chen Z-S. The development of anticancer ruthenium(ii) complexes: From single molecule compounds to nanomaterials. Chem Soc Rev. 2017;46(19):5771–5804. 28654103 10.1039/c7cs00195a PMC5624840

[17] Karges J, Chao H, Gasser G. Critical discussion of the applications of metal complexes for 2-photon photodynamic therapy. J Biol Inorg Chem. 2020;25(8):1035–1050. 33146771 10.1007/s00775-020-01829-5

[18] Liu J, Zhang C, Rees TW, Ke L, Ji L, Chao H. Harnessing ruthenium(II) as photodynamic agents: Encouraging advances in cancer therapy. Coord Chem Rev. 2018;363:17–28.

[19] Zhang L, Montesdeoca N, Karges J, Xiao H. Immunogenic cell death inducing metal complexes for cancer therapy. Angew Chem Int Ed. 2023;62(21):Article e202300662. 10.1002/anie.202300662 36807420

[20] Shum J, Leung PK-K, Lo KK-W. Luminescent ruthenium(II) polypyridine complexes for a wide variety of biomolecular and cellular applications. Inorg Chem. 2019;58(4):2231–2247. 30693762 10.1021/acs.inorgchem.8b02979

[21] Karges J. Combining inorganic chemistry and biology: The underestimated potential of metal complexes in medicine. Chembiochem. 2020;21(21):3044–3046. 32896976 10.1002/cbic.202000397

[22] Karges J, Stokes RW, Cohen SM. Photorelease of a metal-binding pharmacophore from a Ru(ii) polypyridine complex. Dalton Trans. 2021;50(8):2757–2765. 33564808 10.1039/d0dt04290k PMC7944940

[23] Knoll JD, Turro C. Control and utilization of ruthenium and rhodium metal complex excited states for photoactivated cancer therapy. Coord Chem Rev. 2015;282–283:110–126. 10.1016/j.ccr.2014.05.018 PMC4343038 25729089

[24] Karges J, Stokes RW, Cohen SM. Metal complexes for therapeutic applications. Trends Chem. 2021;3(7):523–534. 35966501 10.1016/j.trechm.2021.03.006 PMC9374106

[25] Lee SY, Kim CY, Nam TG. Ruthenium complexes as anticancer agents: A brief history and perspectives. Drug Des Devel Ther. 2020;14:5375–5392. 10.2147/DDDT.S275007 PMC7721113 33299303

[26] Karges J, Kuang S, Ong YC, Chao H, Gasser G. One- and two-photon phototherapeutic effects of RuII polypyridine complexes in the hypoxic centre of large multicellular tumor spheroids and tumor-bearing mice*. Chem Eur J. 2021;27(1):362–370. 32716591 10.1002/chem.202003486

[27] Liu J, Lai H, Xiong Z, Chen B, Chen T. Functionalization and cancer-targeting design of ruthenium complexes for precise cancer therapy. Chem Commun. 2019;55(67):9904–9914. 10.1039/c9cc04098f 31360938

[28] Karges J, Blacque O, Goldner P, Chao H, Gasser G. Towards long wavelength absorbing photodynamic therapy photosensitizers via the extension of a [Ru(bipy)3]2+ core. Eur J Inorg Chem. 2019;2019(32):3704–3712.

[29] Monro S, Colón KL, Yin H, Roque J, Konda P, Gujar S, Thummel RP, Lilge L, Cameron CG, McFarland SA, et al. Transition metal complexes and photodynamic therapy from a tumor-centered approach: Challenges, opportunities, and highlights from the development of TLD1433. Chem Rev. 2019;119(2):797–828. 30295467 10.1021/acs.chemrev.8b00211 PMC6453754

[30] Karges J. Clinical development of metal complexes as photosensitizers for photodynamic therapy of cancer. Angew Chem Int Ed. 2022;61(5):Article e202112236. 10.1002/anie.202112236 34748690

[31] Zamora A, Gandioso A, Massaguer A, Buenestado S, Calvis C, Hernández JL, Mitjans F, Rodríguez V, Ruiz J, Marchán V. Toward angiogenesis inhibitors based on the conjugation of organometallic platinum(II) complexes to RGD peptides. ChemMedChem. 2018;13(17):1755–1762. 29932312 10.1002/cmdc.201800282

[32] Novohradsky V, Zamora A, Gandioso A, Brabec V, Ruiz J, Marchán V. Somatostatin receptor-targeted organometallic iridium(iii) complexes as novel theranostic agents. Chem Commun. 2017;53(40):5523–5526. 10.1039/c7cc01946g 28466888

[33] Barragán F, Carrion-Salip D, Gómez-Pinto I, González-Cantó A, Sadler PJ, de Llorens R, Moreno V, González C, Massaguer A, Marchán V. Somatostatin subtype-2 receptor-targeted metal-based anticancer complexes. Bioconjug Chem. 2012;23(9):1838–1855. 22871231 10.1021/bc300173h

[34] Wang T, Zabarska N, Wu Y, Lamla M, Fischer S, Monczak K, Ng DYW, Rau S, Weil T. Receptor selective ruthenium-somatostatin photosensitizer for cancer targeted photodynamic applications. Chem Commun. 2015;51(63):12552–12555. 10.1039/c5cc03473f 26153573

[35] Barragán F, López-Senín P, Salassa L, Betanzos-Lara S, Habtemariam A, Moreno V, Sadler PJ, Marchán V. Photocontrolled DNA binding of a receptor-targeted organometallic ruthenium(II) complex. J Am Chem Soc. 2011;133(35):14098–14108. 21797210 10.1021/ja205235m

[36] Silva MJSA, Vinck R, Wang Y, Saubaméa B, Tharaud M, Dominguez-Jurado E, Karges J, Gois PMP, Gasser G. Towards selective delivery of a ruthenium(II) polypyridyl complex-containing bombesin conjugate into cancer cells. Chembiochem. 2023;24(4):Article e202200647. 36479913 10.1002/cbic.202200647

[37] Huang Y, Huang W, Chan L, Zhou B, Chen T. A multifunctional DNA origami as carrier of metal complexes to achieve enhanced tumoral delivery and nullified systemic toxicity. Biomaterials. 2016;103:183–196. 27388944 10.1016/j.biomaterials.2016.06.053

[38] Zhu X, Zhou H, Liu Y, Wen Y, Wei C, Yu Q, Liu J. Transferrin/aptamer conjugated mesoporous ruthenium nanosystem for redox-controlled and targeted chemo-photodynamic therapy of glioma. Acta Biomater. 2018;82:143–157. 30316026 10.1016/j.actbio.2018.10.012

[39] McKenzie LK, Flamme M, Felder PS, Karges J, Bonhomme F, Gandioso A, Malosse C, Gasser G, Hollenstein M. A ruthenium–oligonucleotide bioconjugated photosensitizing aptamer for cancer cell specific photodynamic therapy. RSC Chem Biol. 2022;3(1):85–95. 35128412 10.1039/d1cb00146a PMC8729177

[40] Lameijer LN, Hopkins SL, Brevé TG, Askes SHC, Bonnet S. D- versus l-glucose conjugation: Mitochondrial targeting of a light-activated dual-mode-of-action ruthenium-based anticancer prodrug. Chem Eur J. 2016;22(51):18484–18491. 27859843 10.1002/chem.201603066 PMC5214309

[41] Fu H-G, Chen Y, Yu Q, Liu Y. A tumor-targeting Ru/polysaccharide/protein supramolecular assembly with high photodynamic therapy ability. Chem Commun. 2019;55(21):3148–3151. 10.1039/c8cc09964b 30801078

[42] Kaspler P, Lazic S, Forward S, Arenas Y, Mandel A, Lilge L. A ruthenium(ii) based photosensitizer and transferrin complexes enhance photo-physical properties, cell uptake, and photodynamic therapy safety and efficacy. Photochem Photobiol Sci. 2016;15(4):481–495. 26947517 10.1039/c5pp00450k

[43] Chakrabortty S, Agrawalla BK, Stumper A, Vegi NM, Fischer S, Reichardt C, Kögler M, Dietzek B, Feuring-Buske M, Buske C, et al. Mitochondria targeted protein-ruthenium photosensitizer for efficient photodynamic applications. J Am Chem Soc. 2017;139(6):2512–2519. 28097863 10.1021/jacs.6b13399 PMC5588099

[44] Paul S, Kundu P, Bhattacharyya U, Garai A, Maji RC, Kondaiah P, Chakravarty AR. Ruthenium(II) conjugates of boron-dipyrromethene and biotin for targeted photodynamic therapy in red light. Inorg Chem. 2020;59(1):913–924. 31825210 10.1021/acs.inorgchem.9b03178

[45] Xiang H-J, Deng Q, An L, Guo M, Yang S-P, Liu J-G. Tumor cell specific and lysosome-targeted delivery of nitric oxide for enhanced photodynamic therapy triggered by 808 nm near-infrared light. Chem Commun. 2016;52(1):148–151. 10.1039/c5cc07006f 26503188

[46] Zhao X, Li M, Sun W, Fan J, Du J, Peng X. An estrogen receptor targeted ruthenium complex as a two-photon photodynamic therapy agent for breast cancer cells. Chem Commun. 2018;54(51):7038–7041. 10.1039/c8cc03786h 29873358

[47] Du E, Hu X, Roy S, Wang P, Deasy K, Mochizuki T, Zhang Y. Taurine-modified Ru(ii)-complex targets cancerous brain cells for photodynamic therapy. Chem Commun. 2017;53(44):6033–6036. 10.1039/c7cc03337k 28524186

[48] Oliveira G, Gouveia FS, Pinheiro AA, do Nascimento Neto LG, de Vasconcelos MA, Teixeira EH, Gondim ACS, Lopes LGF, de Carvalho IMM, Sousa EHS, et al. An anthracene-pendant ruthenium(ii) complex conjugated to a biotin anchor, an essential handle for photo-induced anti-cancer activity. New J Chem. 2020;44(16):6610–6622.

[49] Jakubaszek M, Rossier J, Karges J, Delasoie J, Goud B, Gasser G, Zobi F. Evaluation of the potential of cobalamin derivatives bearing Ru(II) polypyridyl complexes as photosensitizers for photodynamic therapy. Helv Chim Acta. 2019;102(7):Article e1900104.

[50] Pye H, Stamati I, Yahioglu G, Butt MA, Deonarain M. Antibody-directed phototherapy (ADP). Antibodies. 2013;2(2):270–305.

[51] Karges J, Jakubaszek M, Mari C, Zarschler K, Goud B, Stephan H, Gasser G. Synthesis and characterization of an epidermal growth factor receptor-selective RuII polypyridyl–nanobody conjugate as a photosensitizer for photodynamic therapy. Chembiochem. 2020;21(4):531–542. 31339225 10.1002/cbic.201900419 PMC7065149

[52] Sun D, Liu Y, Yu Q, Zhou Y, Zhang R, Chen X, Hong A, Liu J. The effects of luminescent ruthenium(II) polypyridyl functionalized selenium nanoparticles on bFGF-induced angiogenesis and AKT/ERK signaling. Biomaterials. 2013;34(1):171–180. 23059005 10.1016/j.biomaterials.2012.09.031

[53] Liu T, Zeng L, Jiang W, Fu Y, Zheng W, Chen T. Rational design of cancer-targeted selenium nanoparticles to antagonize multidrug resistance in cancer cells. Nanomedicine. 2015;11(4):947–958. 25680543 10.1016/j.nano.2015.01.009

[54] Sun D, Liu Y, Yu Q, Qin X, Yang L, Zhou Y, Chen L, Liu J. Inhibition of tumor growth and vasculature and fluorescence imaging using functionalized ruthenium-thiol protected selenium nanoparticles. Biomaterials. 2014;35(5):1572–1583. 24268198 10.1016/j.biomaterials.2013.11.007

[55] Wumaier M, Yao T-M, Hu X-C, Hu Z-A, Shi S. Luminescent Ru(ii)-thiol modified silver nanoparticles for lysosome targeted theranostics. Dalton Trans. 2019;48(28):10393–10397. 31162516 10.1039/c9dt00878k

[56] Zhang P, Wang J, Huang H, Chen H, Guan R, Chen Y, Ji L, Chao H. RuNH2@AuNPs as two-photon luminescent probes for thiols in living cells and tissues. Biomaterials. 2014;35(32):9003–9011. 25103232 10.1016/j.biomaterials.2014.07.021

[57] Elmes RBP, Orange KN, Cloonan SM, Williams DC, Gunnlaugsson T. Luminescent ruthenium(II) polypyridyl functionalized gold nanoparticles; their DNA binding abilities and application as cellular imaging agents. J Am Chem Soc. 2011;133(40):15862–15865. 21923121 10.1021/ja2061159

[58] Rogers NJ, Claire S, Harris RM, Farabi S, Zikeli G, Styles IB, Hodges NJ, Pikramenou Z. High coating of Ru(ii) complexes on gold nanoparticles for single particle luminescence imaging in cells. Chem Commun. 2014;50(5):617–619. 10.1039/c3cc47606e 24281162

[59] Frasconi M, Liu Z, Lei J, Wu Y, Strekalova E, Malin D, Ambrogio MW, Chen X, Botros YY, Cryns VL, et al. Photoexpulsion of surface-grafted ruthenium complexes and subsequent release of cytotoxic cargos to cancer cells from mesoporous silica nanoparticles. J Am Chem Soc. 2013;135(31):11603–11613. 23815127 10.1021/ja405058y PMC4086662

[60] Knežević NŽ, Stojanovic V, Chaix A, Bouffard E, Cheikh KE, Morère A, Maynadier M, Lemercier G, Garcia M, Gary-Bobo M, et al. Ruthenium(ii) complex-photosensitized multifunctionalized porous silicon nanoparticles for two-photon near-infrared light responsive imaging and photodynamic cancer therapy. J Mater Chem B. 2016;4(7):1337–1342. 32262989 10.1039/c5tb02726h

[61] He L, Huang Y, Zhu H, Pang G, Zheng W, Wong Y-S, Chen T. Cancer-targeted monodisperse mesoporous silica nanoparticles as carrier of ruthenium polypyridyl complexes to enhance theranostic effects. Adv Funct Mater. 2014;24(19):2754–2763.

[62] Ellahioui Y, Patra M, Mari C, Kaabi R, Karges J, Gasser G, Gómez-Ruiz S. Mesoporous silica nanoparticles functionalised with a photoactive ruthenium(ii) complex: Exploring the formulation of a metal-based photodynamic therapy photosensitiser. Dalton Trans. 2019;48(18):5940–5951. 30209497 10.1039/c8dt02392a

[63] Wen J, Yan H, Xia P, Xu Y, Li H, Sun S. Mesoporous silica nanoparticles-assisted ruthenium(II) complexes for live cell staining. SCIENCE CHINA Chem. 2017;60(6):799–805.

[64] Karges J, Díaz-García D, Prashar S, Gómez-Ruiz S, Gasser G. Ru(II) polypyridine complex-functionalized mesoporous silica nanoparticles as photosensitizers for cancer targeted photodynamic therapy. ACS Appl Bio Mater. 2021;4(5):4394–4405. 10.1021/acsabm.1c00151 35006851

[65] Shi H, Fang T, Tian Y, Huang H, Liu Y. A dual-fluorescent nano-carrier for delivering photoactive ruthenium polypyridyl complexes. J Mater Chem B. 2016;4(27):4746–4753. 32263248 10.1039/c6tb01070a

[66] Chen Y, Jiang G, Zhou Q, Zhang Y, Li K, Zheng Y, Zhang B, Wang X. An upconversion nanoparticle/Ru(ii) polypyridyl complex assembly for NIR-activated release of a DNA covalent-binding agent. RSC Adv. 2016;6(28):23804–23808.

[67] Meijer MS, Natile MM, Bonnet S. 796 nm activation of a photocleavable ruthenium(II) complex conjugated to an upconverting nanoparticle through two phosphonate groups. Inorg Chem. 2020;59(20):14807–14818. 32167752 10.1021/acs.inorgchem.0c00043 PMC7581297

[68] Zhang D-Y, Zheng Y, Tan C-P, Sun J-H, Zhang W, Ji L-N, Mao Z-W. Graphene oxide decorated with Ru(II)–polyethylene glycol complex for lysosome-targeted imaging and photodynamic/photothermal therapy. ACS Appl Mater Interfaces. 2017;9(8):6761–6771. 28150943 10.1021/acsami.6b13808

[69] Wang N, Feng Y, Zeng L, Zhao Z, Chen T. Functionalized multiwalled carbon nanotubes as carriers of ruthenium complexes to antagonize cancer multidrug resistance and radioresistance. ACS Appl Mater Interfaces. 2015;7(27):14933–14945. 26107995 10.1021/acsami.5b03739

[70] Zhang P, Huang H, Huang J, Chen H, Wang J, Qiu K, Zhao D, Ji L, Chao H. Noncovalent ruthenium(II) complexes–single-walled carbon nanotube composites for bimodal photothermal and photodynamic therapy with near-infrared irradiation. ACS Appl Mater Interfaces. 2015;7(41):23278–23290. 26430876 10.1021/acsami.5b07510

[71] Zhang W, Li B, Ma H, Zhang L, Guan Y, Zhang Y, Zhang X, Jing P, Yue S. Combining ruthenium(II) complexes with metal–organic frameworks to realize effective two-photon absorption for singlet oxygen generation. ACS Appl Mater Interfaces. 2016;8(33):21465–21471. 27483010 10.1021/acsami.6b05817

[72] Chen R, Zhang J, Chelora J, Xiong Y, Kershaw SV, Li KF, Lo P-K, Cheah KW, Rogach AL, Zapien JA, et al. Ruthenium(II) complex incorporated UiO-67 metal–organic framework nanoparticles for enhanced two-photon fluorescence imaging and photodynamic cancer therapy. ACS Appl Mater Interfaces. 2017;9(7):5699–5708. 28121418 10.1021/acsami.6b12469

[73] Chen G, Roy I, Yang C, Prasad PN. Nanochemistry and nanomedicine for nanoparticle-based diagnostics and therapy. Chem Rev. 2016;116(5):2826–2885. 26799741 10.1021/acs.chemrev.5b00148

[74] Liang G, Sadhukhan T, Banerjee S, Tang D, Zhang H, Cui M, Montesdeoca N, Karges J, Xiao H. Reduction of platinum(IV) prodrug hemoglobin nanoparticles with deeply penetrating ultrasound radiation for tumor-targeted therapeutically enhanced anticancer therapy. Angew Chem Int Ed. 2023;62(22):Article e202301074. 10.1002/anie.202301074 36961095

[75] De Jong WH, Borm PJA. Drug delivery and nanoparticles: Applications and hazards. Int J Nanomedicine. 2008;3(2):133–149. 18686775 10.2147/ijn.s596 PMC2527668

[76] Cho K, Wang X, Nie S, Chen Z, Shin DM. Therapeutic nanoparticles for drug delivery in cancer. Clin Cancer Res. 2008;14(5):1310–1316. 18316549 10.1158/1078-0432.CCR-07-1441

[77] Wang B, Tang D, Karges J, Cui M, Xiao H. A NIR-II fluorescent polybodipy delivering cationic Pt-NHC with type II immunogenic cell death for combined chemotherapy and photodynamic immunotherapy. Adv Funct Mater. 2023;Article 2214824.

[78] Ma P, Xiao H, Li C, Dai Y, Cheng Z, Hou Z, Lin J, Xiao H, Li C, Dai Y, et al. Inorganic nanocarriers for platinum drug delivery. Mater Today. 2015;18(10):554–564.

[79] Langer R, Tirrell DA. Designing materials for biology and medicine. Nature. 2004;428(6982):487–492. 15057821 10.1038/nature02388

[80] Yu Y, Bu F, Zhou H, Wang Y, Cui J, Wang X, Nie G, Xiao H. Biosafety materials: An emerging new research direction of materials science from the COVID-19 outbreak. Mater Chem Front. 2020;4(7):1930–1953.

[81] Karges J. Combination of chemistry and material science to overcome health problems. Biosaf Health. 2022;4(2):64–65. 35284813 10.1016/j.bsheal.2022.03.004 PMC8906073

[82] Portney NG, Ozkan M. Nano-oncology: Drug delivery, imaging, and sensing. Anal Bioanal Chem. 2006;384(3):620–630. 16440195 10.1007/s00216-005-0247-7

[83] Feng T, Karges J, Liao X, Ji L, Chao H. Engineered exosomes as a natural nanoplatform for cancer targeted delivery of metal-based drugs. Coord Chem Rev. 2022;454:Article 214325.

[84] Svenson S. Dendrimers as versatile platform in drug delivery applications. Eur J Pharm Biopharm. 2009;71(3):445–462. 18976707 10.1016/j.ejpb.2008.09.023

[85] Gao Y, Zhang H, Tang L, Li F, Yang L, Xiao H, Karges J, Huang W, Zhang W, Liu C. Cancer nanobombs delivering artoxplatin with a polyigniter bearing hydrophobic ferrocene units upregulate PD-L1 expression and stimulate stronger anticancer immunity. Adv Sci. 2023;Article 2300806. 10.1002/advs.202300806 PMC10811492 37166035

[86] Chen W-H, Chen Q-W, Chen Q, Cui C, Duan S, Kang Y, Liu Y, Liu Y, Muhammad W, Shao S, et al. Biomedical polymers: Synthesis, properties, and applications. Sci China Chem. 2022;65(6):1010–1075. 35505924 10.1007/s11426-022-1243-5 PMC9050484

[87] Liechty WB, Kryscio DR, Slaughter BV, Peppas NA. Polymers for drug delivery systems. Annu Rev Chem Biomol Eng. 2010;1(1):149–173. 22432577 10.1146/annurev-chembioeng-073009-100847 PMC3438887

[88] Hofmann D, Entrialgo-Castaño M, Kratz K, Lendlein A. Knowledge-based approach towards hydrolytic degradation of polymer-based biomaterials. Adv Mater. 2009;21(32-33):3237–3245. 20882494 10.1002/adma.200802213

[89] Nair LS, Laurencin CT. Biodegradable polymers as biomaterials. Prog Polym Sci. 2007;32(8):762–798.

[90] Bader H, Ringsdorf H, Schmidt B. Watersoluble polymers in medicine. Angew Makromol Chem. 1984;123(1):457–485.

[91] Kopecek J. Soluble biomedical polymers. Polym Med. 1977;7(3):191–221. 593972

[92] Yokoyama M. Clinical applications of polymeric micelle carrier systems in chemotherapy and image diagnosis of solid tumors. J Exp Clin Med. 2011;3(4):151–158.

[93] Soo Choi H, Liu W, Misra P, Tanaka E, Zimmer JP, Itty Ipe B, Bawendi MG, Frangioni JV. Renal clearance of quantum dots. Nat Biotechnol. 2007;25(10):1165–1170. 17891134 10.1038/nbt1340 PMC2702539

[94] Nishiyama N, Kataoka K. Current state, achievements, and future prospects of polymeric micelles as nanocarriers for drug and gene delivery. Pharmacol Ther. 2006;112(3):630–648. 16815554 10.1016/j.pharmthera.2006.05.006

[95] Torchilin VP. Structure and design of polymeric surfactant-based drug delivery systems. J Control Release. 2001;73(2):137–172. 11516494 10.1016/s0168-3659(01)00299-1

[96] Maeda H, Bharate GY, Daruwalla J. Polymeric drugs for efficient tumor-targeted drug delivery based on EPR-effect. Eur J Pharm Biopharm. 2009;71(3):409–419. 19070661 10.1016/j.ejpb.2008.11.010

[97] Wei D, Huang Y, Wang B, Ma L, Karges J, Xiao H. Photo-reduction with NIR light of nucleus-targeting PtIV nanoparticles for combined tumor-targeted chemotherapy and photodynamic immunotherapy. Angew Chem Int Ed. 2022;61(20):Article e202201486. 10.1002/anie.202201486 35212437

[98] Ulbrich K, Holá K, Šubr V, Bakandritsos A, Tuček J, Zbořil R. Targeted drug delivery with polymers and magnetic nanoparticles: Covalent and noncovalent approaches, release control, and clinical studies. Chem Rev. 2016;116(9):5338–5431. 27109701 10.1021/acs.chemrev.5b00589

[99] Yeo Y, Park K. Control of encapsulation efficiency and initial burst in polymeric microparticle systems. Arch Pharm Res. 2004;27(1):1–12. 14969330 10.1007/BF02980037

[100] Gao X, Lei G, Wang B, Deng Z, Karges J, Xiao H, Tan D. Encapsulation of platinum prodrugs into PC7A polymeric nanoparticles combined with immune checkpoint inhibitors for therapeutically enhanced multimodal chemotherapy and immunotherapy by activation of the STING pathway. Adv Sci. 2023;10(4):Article 2205241. 10.1002/advs.202205241 PMC9896041 36504435

[101] Li Q, Li X, Zhao C. Strategies to obtain encapsulation and controlled release of small hydrophilic molecules. Front Bioeng Biotechnol. 2020;8:Article 437. 32478055 10.3389/fbioe.2020.00437 PMC7237580

[102] Liu P, Chen G, Zhang J. A review of liposomes as a drug delivery system: Current status of approved products, regulatory environments, and future perspectives. Molecules. 2022;27(4):1372. 35209162 10.3390/molecules27041372 PMC8879473

[103] Machtakova M, Thérien-Aubin H, Landfester K. Polymer nano-systems for the encapsulation and delivery of active biomacromolecular therapeutic agents. Chem Soc Rev. 2022;51(1):128–152. 34762084 10.1039/d1cs00686j

[104] Chan L, Huang Y, Chen T. Cancer-targeted tri-block copolymer nanoparticles as payloads of metal complexes to achieve enhanced cancer theranosis. J Mater Chem B. 2016;4(26):4517–4525. 32263394 10.1039/c6tb00514d

[105] Bœuf G, Roullin GV, Moreau J, Van Gulick L, Zambrano Pineda N, Terryn C, Ploton D, Andry MC, Chuburu F, Dukic S, et al. Encapsulated ruthenium(II) complexes in biocompatible poly(d,l-lactide-co-glycolide) nanoparticles for application in photodynamic therapy. ChemPlusChem. 2014;79(1):171–180. 31986758 10.1002/cplu.201300242

[106] Karges J, Chao H, Gasser G. Synthesis, characterization, and biological evaluation of the polymeric encapsulation of a ruthenium(II) polypyridine complex with Pluronic F-127/Poloxamer-407 for photodynamic therapy applications. Eur J Inorg Chem. 2020;2020(34):3242–3248.

[107] Karges J, Heinemann F, Jakubaszek M, Maschietto F, Subecz C, Dotou M, Vinck R, Blacque O, Tharaud M, Goud B, et al. Rationally designed long-wavelength absorbing Ru(II) polypyridyl complexes as photosensitizers for photodynamic therapy. J Am Chem Soc. 2020;142(14):6578–6587. 32172564 10.1021/jacs.9b13620

[108] Karges J, Tharaud M, Gasser G. Polymeric encapsulation of a Ru(II)-based photosensitizer for folate-targeted photodynamic therapy of drug resistant cancers. J Med Chem. 2021;64(8):4612–4622. 33818111 10.1021/acs.jmedchem.0c02006

[109] Karges J, Li J, Zeng L, Chao H, Gasser G. Polymeric encapsulation of a ruthenium polypyridine complex for tumor targeted one- and two-photon photodynamic therapy. ACS Appl Mater Interfaces. 2020;12(49):54433–54444. 33238711 10.1021/acsami.0c16119

[110] Dickerson M, Howerton B, Bae Y, Glazer E. Light-sensitive ruthenium complex-loaded cross-linked polymeric nanoassemblies for the treatment of cancer. J Mater Chem B. 2016;4(3):394–408. 26855780 10.1039/C5TB01613D PMC4739781

[111] Ringsdorf H. Structure and properties of pharmacologically active polymers. J Polym Sci Polym Symp. 1975;51(1):135–153.

[112] Sun W, Parowatkin M, Steffen W, Butt H-J, Mailänder V, Wu S. Ruthenium-containing block copolymer assemblies: Red-light-responsive metallopolymers with tunable nanostructures for enhanced cellular uptake and anticancer phototherapy. Adv Healthc Mater. 2016;5(4):467–473. 26680371 10.1002/adhm.201500827

[113] Sun W, Wen Y, Thiramanas R, Chen M, Han J, Gong N, Wagner M, Jiang S, Meijer MS, Bonnet S, et al. Red-light-controlled release of drug–Ru complex conjugates from metallopolymer micelles for phototherapy in hypoxic tumor environments. Adv Funct Mater. 2018;28(39):Article 1804227.

[114] Sun W, Li S, Häupler B, Liu J, Jin S, Steffen W, Schubert US, Butt H-J, Liang X-J, Wu S. An amphiphilic ruthenium Polymetallodrug for combined photodynamic therapy and photochemotherapy in vivo. Adv Mater. 2017;29(6):Article 1603702. 10.1002/adma.201603702 27918115

[115] Zeng X, Wang Y, Han J, Sun W, Butt H-J, Liang X-J, Wu S. Fighting against drug-resistant tumors using a dual-responsive Pt(IV)/Ru(II) bimetallic polymer. Adv Mater. 2020;32(43):Article 2004766. 10.1002/adma.202004766 32964540

[116] Maggioni D, Fenili F, D’Alfonso L, Donghi D, Panigati M, Zanoni I, Marzi R, Manfredi A, Ferruti P, D’Alfonso G, et al. Luminescent rhenium and ruthenium complexes of an amphoteric poly(amidoamine) functionalized with 1,10-phenanthroline. Inorg Chem. 2012;51(23):12776–12788. 23151014 10.1021/ic301616b

[117] Mascheroni L, Dozzi MV, Ranucci E, Ferruti P, Francia V, Salvati A, Maggioni D. Tuning polyamidoamine design to increase uptake and efficacy of ruthenium complexes for photodynamic therapy. Inorg Chem. 2019;58(21):14586–14599. 31618015 10.1021/acs.inorgchem.9b02245

[118] Soliman N, McKenzie LK, Karges J, Bertrand E, Tharaud M, Jakubaszek M, Guérineau V, Goud B, Hollenstein M, Gasser G, et al. Ruthenium-initiated polymerization of lactide: A route to remarkable cellular uptake for photodynamic therapy of cancer. Chem Sci. 2020;11(10):2657–2663. 34084324 10.1039/c9sc05976h PMC8157674

[119] António JPM, Gandioso A, Nemati F, Soliman N, Vinck R, Sun F, Robert C, Burckel P, Decaudin D, Thomas CM, et al. Polymeric encapsulation of a ruthenium(ii) polypyridyl complex: From synthesis to in vivo studies against high-grade epithelial ovarian cancer. Chem Sci. 2023;14(2):362–371. 36687351 10.1039/d2sc05693c PMC9811505

[120] Allen TM, Cullis PR. Liposomal drug delivery systems: From concept to clinical applications. Adv Drug Deliv Rev. 2013;65(1):36–48. 23036225 10.1016/j.addr.2012.09.037

[121] Lian T, Ho RJY. Trends and developments in liposome drug delivery systems. J Pharm Sci. 2001;90(6):667–680. 11357170 10.1002/jps.1023

[122] Shen J, Kim H-C, Wolfram J, Mu C, Zhang W, Liu H, Xie Y, Mai J, Zhang H, Li Z, et al. A liposome encapsulated ruthenium polypyridine complex as a theranostic platform for triple-negative breast cancer. Nano Lett. 2017;17(5):2913–2920. 28418672 10.1021/acs.nanolett.7b00132 PMC5484597

[123] Askes SHC, Bahreman A, Bonnet S. Activation of a photodissociative ruthenium complex by triplet–triplet annihilation upconversion in liposomes. Angew Chem Int Ed. 2014;53(4):1029–1033. 10.1002/anie.201309389 24339049

